# Integrating the smoke-free app into a multicomponent intervention for people with mental health conditions who smoke: a short report of a service-improvement project

**DOI:** 10.1136/bmjph-2025-002740

**Published:** 2025-10-31

**Authors:** Emily Shoesmith, Lisa Huddlestone, Jodi Pervin, Petal Petersen Williams, Jennifer Sweetman, Kerry Johns, David Crane, Louise Ross, Elena Ratschen

**Affiliations:** 1 Department of Health Sciences, University of York, York, UK; 2 Mental Health, Alcohol, Substance Use and Tobacco Research Unit, South African Medical Research Council, Cape Town, South Africa; 3 Institute for Life Course Health Research, Department of Global Health, Stellenbosch University, Stellenbosch, South Africa; 4 The Smoke Free app, London, UK

**Keywords:** Mental Health, Community Health, Health Services Accessibility, Public Health

## Abstract

**Background:**

Evidence on designing and testing digital interventions for patients with mental health conditions is emerging. However, little is known about supporting patient needs and mitigating risks when offering smoking cessation apps in this population. We aimed to identify patient support needs and factors influencing patient safety when interacting with the Smoke Free app.

**Methods:**

Workshops and interviews were conducted with members of a patient and public involvement (PPI) panel and mental health professionals (MHPs). Feedback from PPI members was summarised, and MHP interviews were thematically analysed.

**Results:**

Five PPI members and six MHPs participated. PPI members identified support needs for app use, including tailored demonstrations and reassurance about data confidentiality. MHPs identified safety concerns, including potential misuse of the in-app advisors, risky social interactions when using in-app peer support groups and exacerbation of mental health symptoms related to sharing personal information.

**Conclusion:**

An onboarding session, resources and procedures to support the mitigation of key risks were developed to support patients discharged from acute mental health settings when interacting with the Smoke Free app. The next stage is to deliver the multi-component intervention in a randomised controlled feasibility study, including a process evaluation to assess app uptake and engagement.

**WHAT IS ALREADY KNOWN ON THIS TOPIC**

- Research on digital smoking cessation interventions for people with mental health conditions exists, but little is known about their specific support needs and potential safety concerns. While smoking cessation apps can enhance accessibility and engagement, their use and safety in this population remain underexplored.

**WHAT THIS STUDY ADDS**

- This short report identifies key support needs and safety concerns, including the need for tailored onboarding, concerns about data confidentiality and risks related to in-app peer support and advisor interactions.

**HOW THIS STUDY MIGHT AFFECT RESEARCH, PRACTICE OR POLICY**

- By codeveloping solutions such as onboarding procedures and risk mitigation procedures, the findings of this short report have implications for the development of digital health interventions for people with mental health conditions, to facilitate equitable access within this population.

## Introduction

 Tobacco smoking is up to three times more prevalent among individuals with mental health conditions than among those without.^1^ People with mental health conditions who smoke tend to experience higher levels of nicotine dependence, are at higher risk for smoking-related illnesses and have lower long-term quit rates.^1^ Although this population is able^2^ and motivated^3^ to quit smoking, they are less likely to receive the support required than those without mental health conditions.^4^

In the UK National Health Service (NHS), all mental health settings are smokefree and are expected to provide support for tobacco dependence to patients during their admission.^5^ Research demonstrates that patients can successfully remain abstinent during a mental health inpatient stay when behavioural and pharmacological support are provided.^6^ However, the risk of relapse post discharge is high, with one large study finding 76% of individuals relapsed to smoking within 1 day of discharge and over 90% relapsing within 3 days.^7^ Identifying effective interventions to support patients in maintaining or achieving positive changes in smoking behaviour at discharge is an important evidence gap.^8^

A novel evidence-based and theory-based intervention was codesigned to support smoking behaviour change following discharge from a smokefree mental health stay and is reported in detail elsewhere.^9^ The intervention, delivered by a mental health professional (MHP) with enhanced skills in supporting people with mental health conditions to change their smoking behaviour, includes: (1) predischarge reflection, evaluation and goal setting session(s); (2) a resource folder providing practical and motivational content; (3) tailored behavioural support calls with tapering intensity over a period of 12 weeks; (4) motivational text messaging; (5) a moderated peer support group.

### The Smoke Free app

The Smoke Free app is an evidence-based and theory-based smartphone application which includes behaviour change techniques (ie, structured strategies, such as goal setting, self-monitoring and feedback) that are known to improve an individual’s chances of quitting smoking.^10^ The Smoke Free app is informed by behaviour change theory^11^ and directly aligns with the standards of smoking cessation support defined by the National Centre for Smoking Cessation and Training. Users of the app are able to access daily challenges, personalised progress, health and financial trackers and a private Facebook group for peer support at no cost. For a paid subscription, the app provides additional features, including anytime synchronous text-based support from qualified smoking cessation advisors (referred to as advisors).

Following a desk-based review, the app was identified as a potential adjunct to the intervention to ensure seamless behavioural support for patients during the postdischarge period, leading to increased engagement. However, limited research exists on the use of mobile health (m-health) technologies in supporting smoking-related behaviour change in this population. The offer of an evidence-based app is timely, given how digital approaches are diffusing into society,^12^ and presents an opportunity to increase equity of access to technology shown to have the capacity to support positive health behaviour change.^13^ However, it is important to understand and mitigate for the potential vulnerabilities and support needs of individuals with mental health conditions when using and engaging with m-health technologies.^14^ Therefore, this brief report considers factors influencing safeguarding, access and use of m-health technology by adults with mental health conditions and develops solutions to safe engagement. The objectives were to:

1. Identify patient support needs related to app onboarding;
2. Codesign an acceptable user onboarding session to promote and sustain use of the Smoke Free app;
3. Identify patient and app-related factors influencing safety when interacting with the Smoke Free app;
4. Codevelop procedures to support the mitigation of key risks relating to use of the Smoke Free app.

## Methods

This service-improvement project adopted a qualitative design, combining PPI workshops with semi-structured interviews with MHPs. This approach was selected to generate practical, stakeholder-informed insights into support needs, safety concerns and feasible risk-mitigation strategies for integrating the Smoke Free app into a postdischarge intervention.

### Recruitment and procedures

#### Identification of support needs related to app onboarding and development of an onboarding session

Three workshops were conducted with an existing PPI panel. The first workshop provided a detailed overview and demonstration of the Smoke Free app. The second workshop took place 1 week after the first, giving PPI members 7 days to download and explore the app, noting any challenges relating to set-up, use and navigation and how these could be overcome. Based on these insights and potential solutions, a user onboarding session and support resources were developed, in collaboration with the app developers, and presented to the PPI panel in a third workshop. PPI members were asked to share their views and suggestions for refinements. Based on the feedback collected, draft onboarding materials were developed and modified iteratively to reflect PPI members’ suggestions.

PPI involvement was integral to the development process as this project aimed to co-produce an intervention tailored to the needs of people with mental health conditions. Engaging individuals with lived experience ensured that the app onboarding materials and risk-mitigation strategies were acceptable, feasible and relevant to the intended users.

#### Identification of patient and app-related factors influencing safety and development of procedures to mitigate key risks

An academic researcher conducted semistructured interviews with MHPs to explore potential safety issues associated with app use. Participants were purposively recruited via email invitations distributed within a participating NHS Trust in Yorkshire and were eligible if employed in inpatient or community mental health services at that Trust. All interviews were conducted remotely via secure video conferencing by the first author, using a topic guide (online supplemental material 1). From the interviews, potential solutions were identified and shared with an expert group of MHPs, academics and members of the Smoke Free app development team for consideration.

The project was reviewed and received approval as a service-related project by both the participating Trust’s Research and Development department and the University of York Health Sciences Research Governance Committee (reference: HSRGC/2023/546 /A). All participants received information sheets explaining the aims of the project and provided written consent. All participants were compensated with shopping vouchers (£25 per hour).

### Data analysis

Data were analysed by participant group. Feedback provided by PPI members was collated and summarised. Qualitative data from interviews with MHPs were analysed using inductive thematic analysis,^15^ with one researcher generating initial codes and themes, which were then reviewed with the wider team to ensure agreement. Reflexivity was addressed through team discussions to minimise the influence of the interviewer’s academic background on data interpretation.

## Results

Five members of an existing PPI panel were recruited and completed three workshops. Participants had either lived experience of acute adult mental health inpatient services and current or previous tobacco smoking (n=4) or experience of supporting a relative who smokes and was recently discharged from a mental health inpatient stay (n=1). Six MHPs were recruited and completed interviews. MHPs’ experience ranged from 2.5 years to 20 years within inpatient services (n=2) and community mental health teams (n=4). Interviews with MHPs ranged from 30 min to 45 min.

### Identification of support needs related to app onboarding and development of onboarding session

The onboarding materials were presented to the PPI panel in a third workshop, where members provided additional feedback, leading to further refinements prior to finalisation. Table 1 summarises participants’ experiences, challenges and proposed solutions.

**Table 1:** Key experiences, challenges and respective solutions

Key experience/challenges	Solution
A detailed demonstration of downloading and navigating the app was deemed as beneficial (especially for those who had limited digital skills). Providing supplementary videos and written information for patients to refer to in their own time would also be valuable.	A patient-led detailed demonstration to be provided during the onboarding session (based on individual need and digital literacy). Patients will also be provided with a series of videos focusing on various elements of the app, and a written information pack (using visual screenshots of the app) which they will have access to in their own time post discharge.
Issues around certain acronyms used in the app that patients may not be familiar with.	All acronyms will be spelled out fully, but information will also be provided in the written information pack that covers a list of commonly used acronyms.
Concerns were expressed about data use and confidentiality of users’ information. Specifically, PPI members requested clear reassurance that their personal data would remain confidential.	Information will be provided in the onboarding session to assure patients that all data will be protected. Data entered into the app will only be used for the app team to personalise conversations they have within the 24-hour text-based support. A short video and written information will also be provided to highlight key points in a short and user-friendly manner.
Concerns were expressed around the frequency of push notifications. Specifically, the frequency may become overwhelming for some.	During the onboarding session, supplementary videos and written information will cover initial personalisation of the app and turning off or reducing notifications as desired by patients.
Issues around the use of ‘live advisor’ support. Specifically, some patients may have expectations that the advisor can offer support beyond that of smoking-related support.	Information on the appropriate use of the app and interactions with advisors will be provided in the onboarding session to ensure expectations and boundaries are clear. Specifically, patients will be informed that their primary intervention provider will be their first point of contact and app advisors are available to offer smoking-related advice only. This will be highlighted in the onboarding session and continuously in weekly interactions with the primary intervention provider.
Focus on quantitative data entry (eg, inputting numerical information related to money saving) was considered off-putting by some; preference was for qualitative-based features (eg, writing free-text entries into the diary).	The onboarding session will include information on how to personalise the in-app dashboard, including only the features that work best for each individual, and ensure patients are aware they do not have to use every feature available. A short video and written information will also be provided on how to personalise the app and navigate to favourite features.
Inclusion of personal quitting stories and testimonies of the benefit of using the app would be beneficial.	A member of the existing PPI panel wrote a testimony about their experience of using the app and the associated benefits. This will be available to interested patients during the onboarding session.
Various user-friendly modes of delivery to provide information were discussed, so patients could seek guidance that is best suited to their learning style.	A detailed demonstration and overview will be provided in the onboarding session (as needed), adopting an approach that promotes autonomy rather than instructing the patient. Videos and written information will also be provided so assistance is accessible in a variety of formats.

PPI, patient and public involvement.

### Identification of patient and app-related factors influencing safety and development of procedures to mitigate key risks

Four themes were identified from interviews with MHPs that related to factors influencing the safe use of the app. Only a summary of key themes is provided here due to the short-report format (illustrative quotes are provided in online supplemental material 2).

#### Risks relating to the advisor chat function

MHPs considered the potential for patients to use the in-app advisor chat function for mental healthcare seeking or escalation. Some MHPs also highlighted the potential harm that could result from a lack of awareness of the interaction between changes in smoking behaviours and some psychotropic medications, and the in-app advisor chat function should not be used to seek advice if medication requires altering. They also noted the potential for some patients to develop attachments to specific advisors, which could challenge boundaries and negatively impact mental health if a preferred advisor was unavailable:

#### Interactions, alliances and relationships within the app

MHPs referred to the potential risk of unhelpful social interactions that may occur when using peer support groups or forums available via the app. They reported that this type of support is not always used appropriately in this population and highlighted the requirement for appropriate moderation by experienced app advisors.

#### Potential impact of the app on mental health

While smartphones were considered advantageous in inpatient wards (eg, allowing access to music, media and social networks), app use was considered a potential risk for exacerbating mental health symptoms. For example, MHPs referred to how app use had impacted symptoms such as paranoia and anxiety (eg, when patients may be apprehensive to input personal details).

#### Suggested mitigations to reduce risk

MHPs suggested the need for boundaries and consistency of behavioural expectation and emphasised these should be established with patients prior to them using the app: MHPs also highlighted the need for a standardised and structured protocol to guide advisors in responding to any risk disclosures by patients. Lastly, MHPs proposed that encouraging patients to view the advisors as a team may help to overcome the potential for any risks arising should a preferred advisor be unavailable.

### Development of feasible procedures to support the mitigation of key risks

Procedures developed, in collaboration with the expert panel, to mitigate user-related risks are presented in table 2.

**Table 2:** Procedures to mitigate risks related to use of the Smoke Free app

Mitigation aim	Procedure/action
Provision of information on the appropriate use of the app and interactions with the advisor.	Information on the appropriate use of the app and interactions with the advisors will be provided in the onboarding session to ensure expectations and boundaries are clear. Specifically, patients will be informed their primary intervention provider will be their first point of contact and advisors are available to offer smoking-related advice only. This will be highlighted in the onboarding session and continuously in weekly interactions with the primary intervention provider.
Provision of information to re-direct patients to their mental health team in the case of mental healthcare seeking and/or changes in smoking behaviour.	A standardised script has been developed in collaboration with the clinical co-applicants for advisors to use in the case of any mental healthcare seeking and/or changes in smoking behaviour. This will be provided to patients in the onboarding session so they are aware they will receive a standardised response re-directing them to their designated care team. This script will also be provided in the written information pack.
Evaluation of appropriate use of the advisor function.	The research team will receive information from the app team if a patient has made a disclosure to the advisors, and this will be continuously reviewed on a weekly basis. Checkpoint meetings with clinical co-applicants will be held if disclosures are frequently made to discuss appropriate courses of action.
Supporting advisors to feel confident re-directing patients to mental health services in cases of care-seeking, disclosures or changes in smoking behaviour.	A training session will be provided to the advisors to ensure they are confident in re-directing patients and using the standardised script provided.
Supporting the delivery of consistent whole-team support.	A collaborative, team-based approach will be emphasised during the onboarding session to highlight that the patient will have access to a community of smoking advisors. This will help to establish boundaries from the outset and ensure the patient is aware they will not always be speaking to the same advisor.
Supporting patients to engage safely with online forums.	The online forums are appropriately moderated by an experienced advisor.
Addressing practical and illness-related factors challenging app use.	Patients will be assured in the onboarding session that all data will be protected. Personal data will only be used by the app team to personalise conversations in the 24-hour text-based support feature. Supplementary resources will be provided to patients to highlight these key points.

## Discussion

This report explored integrating the Smoke Free app into a multicomponent intervention for individuals with MHCs who smoke, highlighting the need for tailored onboarding, support and safeguards against risks like app misuse and data concerns.

Digital interventions may offer a promising alternative for smoking cessation in this population,^16^ with apps providing scalability across healthcare settings and potential for improved adherence due to their convenience.^17^ However, a recent review suggests that while many apps exist to support individuals with MHCs, few are accessible or suitable,^18^ highlighting the need for developers and providers to address barriers to usage.^19^

While research has explored the acceptability and feasibility of smoking cessation apps for individuals with MHCs,^20^ little exists on supporting their use. These findings address the unique needs of this population and highlight key safety considerations. By co-designing support resources and risk mitigation procedures, these findings bridge evidence gaps by facilitating our understanding of safe integration of digital health tools in this population.

Future research is crucial to improve the dissemination of effective digital approaches. The next phase involves delivering the multicomponent intervention in a randomised controlled feasibility study, alongside a process evaluation to assess the app’s accessibility.

Within the limits of a short service-improvement report (including a small sample size and generalisability of recruitment from a single NHS Trust), this work contributes to the field by providing practical, co-produced procedures to address safety and accessibility challenges in using digital tools to support smoking cessation for people with MHCs, an area where evidence remains limited.

## Supplementary material

10.1136/bmjph-2025-002740 online supplemental file 1

10.1136/bmjph-2025-002740 online supplemental file 2
