# Supplementary material for: Integrating the smoke-free app into a multicomponent intervention for people with mental health conditions who smoke: a short report of a service-improvement project
Source: BMJ Public Health. 2025 Oct 31;3(2):e002740. doi: 10.1136/bmjph-2025-002740 (PMC12581057; doi:10.1136/bmjph-2025-002740)
Supplement: online supplemental file 1 [file bmjph-3-2-s001.docx]

**Supplementary Material 1.** MHP topic guide

| 1. Introduction | - Thank participant(s) for attending. - Provide outline of the interview and confirm consent for recording. - Obtain participant information:   - Job role   - Length of time in role |
| --- | --- |

| 1. Study purpose and introduction to the App | - Provide a brief overview of the aims of SCEPTRE and the need to include the Smoke Free App. - Briefly detail the functions of the Smoke Free App. - Ask participants to keep these in mind when responding to questions. |
| --- | --- |

| 1. Topics for clinician consideration | 1. General thoughts around the use of smartphone apps to support smoking cessation in people with mental illness.  - Phone ownership / digital literacy/ availability Affordability of data - Population benefits/challenges - Risks associated with the use of apps generally and specifically for people recently discharged from acute MH inpatient admission.  1. Thoughts specifically relating to the risks or safeguarding issues surrounding use of the Smoke Free App in the immediate post discharge period.    - Use of live text chat function    - Deterioration of mental health    - Changes in tobacco smoking by those prescribed clozapine and other antipsychotics with toxicity risks.    - Potential for participants to drop the personalised support with MTS in favour of the app advisors. 2. Thoughts around how safety and safeguarding can be assured in patients using the app in the immediate post discharge period and up to three months post discharge can be minimised.    - Perceived likelihood of risks occurring    - Communication between MTS/researchers and clinical teams in circumstances requiring escalation.    - Advisor actions/protocols for advising participants in distress to refer to their care/crisis plans. Would scripts be useful? |
| --- | --- |

| 1. Close | - Ask participants if they would like to add to or clarify any of their responses. - Ask what influenced the decision to participate in the study. - Would they be interested in taking part in a small focus group to review the finalised safety protocol document. - Thank the participant for their time and request address to send the voucher to. - End the interview. |
| --- | --- |
