# Supplementary material for: Integrating the smoke-free app into a multicomponent intervention for people with mental health conditions who smoke: a short report of a service-improvement project
Source: BMJ Public Health. 2025 Oct 31;3(2):e002740. doi: 10.1136/bmjph-2025-002740 (PMC12581057; doi:10.1136/bmjph-2025-002740)
Supplement: online supplemental file 2 [file bmjph-3-2-s002.docx]

**Supplementary Material 2.** Illustrative quotes

| **Theme** | **Illustrative quotes** |
| --- | --- |
| Risks relating to the advisor chat function | " I have a couple of people on my caseload that would probably…like they’d tell anybody that would listen that they’ve taken an overdose and they won’t have done, and then that would put the professional person at a point where they would have to call an ambulance." P002. |
| Interactions, alliances, and relationships within the app | "How that’s going to be moderated. What information people are going to be sharing. How that’s shared. Whether people start engaging in unhelpful conversations, or making threats" P005_006. |
| Potential impact of the app on mental health | “…for some people having a phone can be stressful and exacerbate their thinking, unusual thinking” P001. |
| Suggested mitigations to reduce risk | "the notion of boundaries for clinicians is incredibly important and being skilfully maintaining your boundaries while maintaining a therapeutic relationship" P001. |
